# Moshi-Moshi - development of an app to help in the treatment of suicidal patients: between training and treatment

**DOI:** 10.1192/j.eurpsy.2026.10303

**Published:** 2026-07-31

**Authors:** R. Calati, Marianna Poli, Federica Turolla, Martina Colognesi, Arian Fooroogh Mand Arabi, Silvia Montesuelli, Erica Ruffilli, Alessandra Villani, Sara Brivio, Valentina Cazzaniga, Fabio Madeddu, Riccardo Nigrelli, Daniela Micucci, Carla Gramaglia, Patrizia Zeppegno

**Affiliations:** Department of Psychology, University of Milan-Bicocca , Milan, Italy

## Abstract

**Background:**

Suicide represents a major public health challenge worldwide. Despite the availability of evidence-based treatments, many individuals at risk face significant barriers to accessing timely, personalized, and continuous psychological support. Digital interventions offer a promising opportunity to expand access to care; however, many currently available tools show limited personalization, variable engagement, and high dropout rates, reducing their potential clinical impact.

**Aims and Methods:**

MoshiMoshi is an innovative project integrating clinical research and digital technology to deliver a tailored, multi-component psychological intervention for individuals experiencing suicidal ideation or with a history of suicide attempts. The program lasts seven weeks and is delivered through a mobile application within a blended-care framework. It combines psychoeducation and experiential exercises informed by cognitive behavioral therapy (CBT), dialectical behavior therapy (DBT), acceptance and commitment therapy (ACT), mindfulness-based approaches, and interpersonal psychotherapy (IPT). All participants complete a core module focused on understanding and managing suicidal ideation. Additional modules—addressing depression and anxiety, burnout, chronic pain, relational difficulties, and lifestyle problems—are assigned based on individualized screening and clinical assessment to ensure a personalized treatment pathway. The intervention is anchored by two in-person clinical sessions dedicated to safety planning at the beginning and relapse prevention at the end of the program, while digital modules are completed autonomously between sessions. Ongoing assessments and daily ecological check-ins are embedded in the app to monitor risk and engagement.

**Results:**

Development and preliminary testing were conducted through two pilot studies involving university students. Pilot 1 and Pilot 2 evaluated feasibility, usability, and acceptability. Satisfaction and usability ratings were high in both studies. Participants particularly appreciated daily monitoring questions and guided meditation exercises. Although engagement fluctuated over time and dropout rates were consistent with those typically reported in digital mental health research, overall feedback supported the intervention’s feasibility. A controlled trial is currently underway to evaluate the efficacy of MoshiMoshi compared with treatment as usual in a clinical sample.

**Conclusions:**

By integrating clinical expertise, individualized module selection, structured safety components, and digital delivery, MoshiMoshi seeks to strengthen suicide prevention efforts through accessible, personalized, and engaging psychological support.

**Disclosure of Interest:**

None Declared

